# The pathway toward practical application of lithium-metal anodes for non-aqueous secondary batteries

**DOI:** 10.1093/nsr/nwac031

**Published:** 2022-02-28

**Authors:** Panlong Li, Zhong Fang, Xiaoli Dong, Congxiao Wang, Yongyao Xia

**Affiliations:** Department of Chemistry and Shanghai Key Laboratory of Molecular Catalysis and Innovative Materials, Institute of New Energy, iChEM (Collaborative Innovation Center of Chemistry for Energy Materials), Fudan University, Shanghai 200433, China; Department of Chemistry and Shanghai Key Laboratory of Molecular Catalysis and Innovative Materials, Institute of New Energy, iChEM (Collaborative Innovation Center of Chemistry for Energy Materials), Fudan University, Shanghai 200433, China; Department of Chemistry and Shanghai Key Laboratory of Molecular Catalysis and Innovative Materials, Institute of New Energy, iChEM (Collaborative Innovation Center of Chemistry for Energy Materials), Fudan University, Shanghai 200433, China; Department of Chemistry and Shanghai Key Laboratory of Molecular Catalysis and Innovative Materials, Institute of New Energy, iChEM (Collaborative Innovation Center of Chemistry for Energy Materials), Fudan University, Shanghai 200433, China; Department of Chemistry and Shanghai Key Laboratory of Molecular Catalysis and Innovative Materials, Institute of New Energy, iChEM (Collaborative Innovation Center of Chemistry for Energy Materials), Fudan University, Shanghai 200433, China

**Keywords:** lithium-metal anode, failure mode, concentration polarization, dead Li

## Abstract

The revolution of automotive vehicles (from petrol vehicles to electric vehicles) has set high demands for the performance of batteries. Lithium-metal batteries (LMBs) show great potential owing to their high energy density but encounter poor cycle life and safety issues. It is of great significance to reveal LMB failure mechanisms and understand their relationship with battery performance. This review presents an overview of the state-of-the-art Li-metal anodes, with an emphasis on two typical failure modes: capacity degradation and dendritic growth of Li metal. The critical correlations between the composition, structure and failure are explained point by point. The chemical and electrochemical stabilities of the lithium anode are discussed. Particularly, for the first time, five types of lithium-metal anodes are classified to develop a comprehensive understanding of LMBs. Furthermore, strategies are suggested to improve the practical performance of LMBs, including material innovation, electrolyte modification and advanced characterization.

## Teaser

The state-of-the-art Li-metal anodes have been well discussed with comprehensive view of Li-metal anodes and their failure modes, which paves a way to better improve the practical lithium metal batteries.

## INTRODUCTION

In the early development of secondary lithium batteries, the electrochemical process at the negative electrode was based on lithium plating/stripping in consideration of designing high-energy-density storage systems, owing to the high capacity and low potential of Li-metal anodes. However, the rapid commercialization of lithium-metal batteries (LMBs) stagnated due to the high reactivity of lithium metal and related interfacial passivation and its instability. The practical application of LMBs eventually ended with several occasional fire accidents proven by intensive reliability tests by the Nippon Electric Company and Mitsui [1]. The safety issues for lithium metal were tactfully circumvented by use of rocking-chair technology, which is well known and commonly used for lithium-ion batteries (LIBs). Carbonaceous material with high reversibility and low voltage was finally selected as an anode material to assemble C/LiCoO_2_ rocking-chair cells by the Sony Corporation in 1991. Benefitting from intercalated graphite materials, rocking-chair batteries avoid the problems associated with Li-metal anodes. The innovation of graphite anodes has accelerated the wide application of LIBs, which can be used on multiple platforms ranging from portable electronic devices to various electrified transportation systems [1]. According to statistical data, 1% of the automotive market consumed 60% of the LIB supply in 2018. The market prospects for electric vehicles (EVs) have attracted the interest of many large companies and laboratories. However, the rapid growth of EVs and portable electric devices calls for next-generation high-energy rechargeable batteries, which has revived interest in the use of high-energy Li-metal anodes as long as safety issues and capacity loss can be addressed [2]. As a result, solutions to the Li-metal problem are urgently needed for the practical use of Li-metal anodes. To seek perfect solutions, the first thing that needs to be determined is the core factors that lead to failure. To detect the failure mechanism, a series of techniques, including physical, chemical and electrochemical characterizations, are proposed.

**Figure 1.**
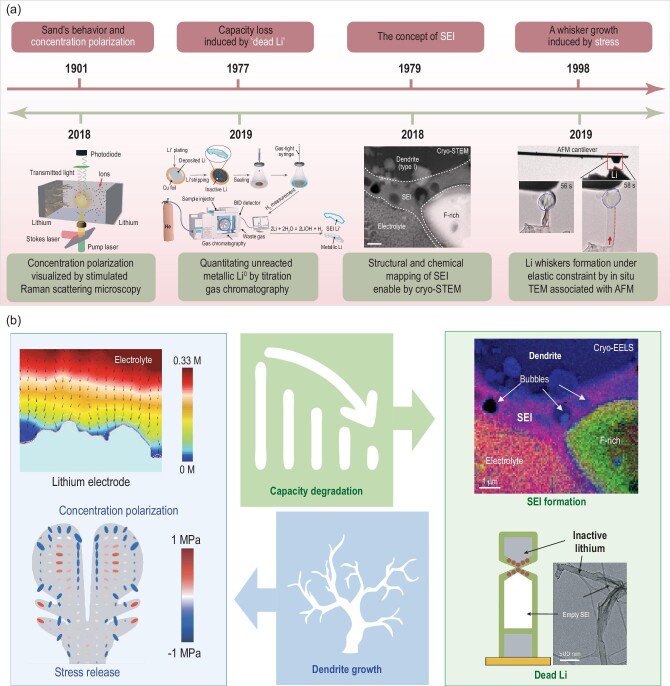
(a) Advanced characterization technologies facilitate the understanding of the lithium-failure mechanism and the confirmation of hypotheses in the past [3–6]. STEM, scanning transmission electron microscopy; AFM, atomic force microscopy. Copyright 2018, Springer Nature Limited; Copyright 2019, Springer Nature Limited; Copyright 2018, Springer Nature Limited; Copyright 2019, Springer Nature Limited. (b) Four main failure reasons of lithium metal in rechargeable lithium-metal batteries (i.e. concentration polarization [3], Copyright 2018, Springer Nature Limited; stress release [7], Copyright 2019, Royal Society of Chemistry; solid-electrolyte interphase (SEI) formation [5], Copyright 2018, Springer Nature Limited; dead Li [8], Copyright 2018, Elsevier).

In recent years, tremendous efforts have been made in the field of characterization technologies, theoretical calculation methods and so on to understand the failure mechanism for lithium metal and promote the progress of practical applications (Fig. 1a) [3–8]. Despite some reviews on this topic, no concise retrospect has been made for the technologies and theories required for understanding the failure mechanism of lithium-metal anodes [9–11]. However, no explicit classification of lithium-metal anodes has been systematically categorized and discussed. To fill this gap, we summarize and review two failure modes for lithium metal and provide new understanding for four major factors responsible for battery failure in rechargeable LMBs (i.e. concentration polarization, stress release, solid-electrolyte interphase (SEI) formation and dead Li) (Fig. 1b). In the following section, we classify and review five kinds of lithium-metal anodes (i.e. stabilized lithium-metal powder (SLMP), stabilized lithium-metal anode (SLMA), deposited lithium-metal anode (DLMA), composite lithium-metal anode (CLMA) and anode-free lithium-metal anode (AFLMA)), according to their preparation methods and application potentials.

## THE FAILURE OF THE LITHIUM-METAL ANODE

During the operation of LMBs, two typical failure modes are reviewed for Li-metal anodes in this section: *short-circuiting* and *fast capacity degradation*, which can be ascribed to dendrite growth and increased inner resistance of LMBs, respectively. Furthermore, dendrite formation is related to both external (such as the ion distribution in the electrolytes) and internal factors (such as the stress distribution and release for lithium electrodeposition), while Li-metal capacity degradation corresponds to hyperactivity with almost all the components in the liquid electrolytes. These side reactions are both beneficial (such as a protective layer for lithium metal) and harmful (such as the consumption of active materials and the formation of ‘dead Li’). We hereby summarize and review the developed insights and theories for the failure mechanism of Li-metal anodes, combined with the understanding and hypotheses in early LMB research.

### The mechanism for dendrite-lithium formation

The inhomogeneous ion distribution in the liquid electrolyte is the primary factor that leads to non-uniform Li-dendrite formation. In addition, the uneven stress distribution and release of deposited Li lead to ramified morphologies. Owing to differences in test conditions, the observed morphologies for the Li dendrites vary in different reports. In this section, we review several reasonable mechanisms that have been observed experimentally and proven theoretically.

#### The inhomogeneous growth induced by concentration polarization

Early investigations started with the relationship between the ion distribution and the electrodeposition behavior. Based on the sulfuric acid and copper sulfate system, Sand explored the concentration at the electrodes in 1901 [12]. It was reported that the concentration of copper can rapidly decline to near zero when applying a high current density. Sufficient copper ions could not be transported to the electrode by diffusion to preserve electroneutrality and mass transfer predominated over charge transfer. The time required for the concentration to decrease to zero at the negative electrode and this kind of process are generally called ‘Sand's time’ and ‘Sand's behavior’, respectively. Inspired by Sand's work, metallic electrodeposits were further investigated in the high-current-density regime. It is widely thought that the cationic concentration is reduced to zero in the vicinity of the negative electrode in Sand's time, which results in a non-classical space-charge region near the deposit and an electric field in the deposited bulk. It is believed that the unstable space charge and electric field result in the growth of metallic dendrites [13]. These models for metal ion electrodeposition were further confirmed via *in situ* observation in symmetrical lithium cells based on PEO-LiTFSI (polyethylene oxide- bistrifluoromethanesulfonimide lithium salt) electrolyte [14]. Moreover, dendrites with different morphologies were also observed in a low-current-density (far below the diffusion-limited current) regime. This might be related to the local inhomogeneities in the vicinity of the electrode, which are induced by the passivation layer or the structure of the electrode.

**Figure 2.**
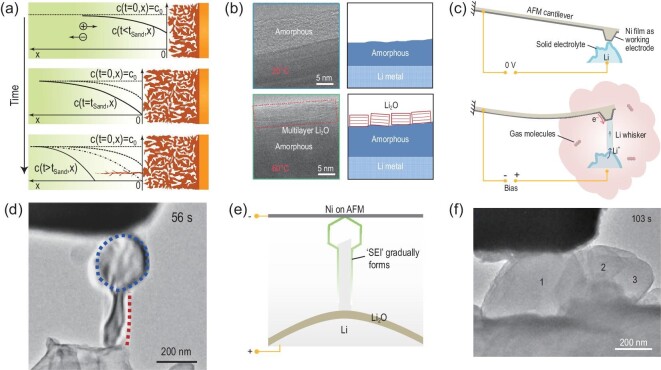
(a) Theoretical interpretation of the growth mechanisms of lithium electrodeposition during concentration polarization [15]. Copyright 2016, Royal Society of Chemistry. (b) Cryo-EM reveals an emergent SEI nanostructure formed at elevated temperature and the corresponding schematics of the observed amorphous SEI structure on a Li particle grown at 20°C and a thicker layered SEI nanostructure on a Li particle grown at 60°C [17]. Copyright 2019, Springer Nature Limited. (c) Schematic of the experimental set-up before Li deposition and a growing Li whisker pushing the atomic force microscopy (AFM) cantilever [6]. Copyright 2019, Springer Nature Limited. (d) Li-whisker formation during electrochemical deposition of Li in a CO_2_ environment [6]. Copyright 2019, Springer Nature Limited. (e) Schematic interpretation of the *in situ* environmental transmission electron microscopy (ETEM) observations in (d). (f) Li-whisker formation during electrochemical deposition of Li in a N_2_ environment [6]. Copyright 2019, Springer Nature Limited.

To understand the process of electrolyte dynamics and electrolyte–electrode interactions, advanced stimulated Raman scattering microscopy has recently been used to visualize the ion distribution in lithium-salt electrolytes [3]. It was revealed that the lithium dendrite growth should be ascribed to the heterogeneity of the local ionic concentration near the negative electrode. Sand's work has had a great influence on investigations of metallic electrodeposition, especially lithium deposition. With *in situ* optical glass capillary cells, mossy and dendritic lithium models were analysed based on the pre- and post-Sand time [15]. The mossy lithium is easily suppressed by a special separator, while dendritic lithium penetrates the separator and finally leads to a short circuit in the cell (Fig. 2a). Therefore, it is suggested that highly concentrated electrolytes (with a long Sand time) and appropriate separators can improve the safety performance of LMBs.

Although a high-current-density regime has been widely considered to exacerbate the growth of dendritic lithium, it was reported that lithium dendrites can be healed by applying a high current density of 15 mA cm^–2^ [16]. Further analysis revealed that healing can be ascribed to accelerated lithium-ion transport by self-heating at a high current density and the corresponding dendrite temperatures were theoretically predicted to range from 60–80°C at this current density. As early as 2002, elevated temperature (60–80°C) had already been proven to improve the cycling stability of deposited Li on nickel substrates, which can form particle-like deposits with a diameter of 100–200 nm with a dense and uniform surface film [18]. It was further proposed that the deposition kinetics can be effectively altered by applying higher temperatures near the anode surface, which favors the formation of a more uniform morphology. Computational investigation of the thermal effect of the behavior of lithium electrodeposition indicated a decrease in the normalized dendrite length with increasing ambient temperature [19]. Recently, the improved performance of deposited lithium at an elevated temperature of 60°C was further investigated via cryo-electron microscopy (EM), revealing a different SEI nanostructure with extra multi-layer Li_2_O on Li particles grown compared to the amorphous nanostructure formed at 20°C (Fig. 2b) [17]. Notably, our group used *in situ* optical microscopy to show that elevated temperature relieves the trend for lithium divarication, but inhomogeneous growth of lithium electrodeposition still exists [20]. Therefore, we proposed the use of a highly concentrated electrolyte accompanied by elevated temperature to realize uniform electrodeposition of lithium.

#### The ramified morphologies resulting from residual stress

Apart from the concentration polarization, the distribution and release of stress play a crucial role in determining the ramified morphologies of dendritic lithium. Similar to electrodeposited Sn, Cu or other metals, the driving force is blamed for the whisker formation of electrodeposited lithium metal [21]. In 1998, a whisker-growth mechanism was proposed in which non-uniformly deposited lithium suffers pressure induced by interface tension throughout the lithium interface and the SEI layer [22]. The deposited lithium breaks and releases the stress for the growth of whiskers. However, it was difficult to prove this plausible conclusion with experiments at that time. Later, a stress-relief mechanism was proposed, which indicated that wrinkling helps reduce the residual stress in plated lithium [23]. The corresponding soft substrate with wrinkling (versus hard Cu foils) was confirmed to release the stress in Li-dendrite growth during electrodeposition, thus proving that the stress-driven dendrite growth model can mitigate lithium whiskers. Nevertheless, although the stress-relief mechanism partly explains the formation of lithium whiskers, a fundamental understanding of various experimentally observable morphologies remains lacking. Researchers have employed one thermodynamically consistent theory and three effects (chemical diffusion, electrodeposition, and elastic and plastic deformation kinetics) to identify six lithium electrodeposition regimes: (i) thermodynamic suppression regime, (ii) incubation regime, (iii) base-controlled regime, (iv) tip-controlled regime, (v) mixed regime and (vi) Sand's regime [7]. They explained the microstructural evolution of lithium electrodeposits, such as plastic flow at the tips, dendrite bifurcation, and bent and kinked morphologies. Moreover, the general sources of mechanical stress in the growth of lithium dendrites were summarized, including adjoining electrodeposits, separators, the cell casing, local volume changes due to the SEI layer and so on.

Depending on the combination of an atomic force microscopy (AFM) cantilever and *in situ* environmental transmission electron microscopy (ETEM) (Fig. 2c), it was proposed that the retarded surface transport of Li in the SEI layer plays a decisive role in forming steady morphologies during lithium plating [6]. The evolution process of the whisker lithium morphology starts from a non-directional mode to a directional mode, which was interpreted from the dynamics of the sluggish transport behavior of lithium in the initial SEI and the thermodynamic mechanism of the minimum surface energy. The specific function of the component in the SEI layer was well analysed. Lithium electrodeposition was conducted in N_2_ and CO_2_ gas atmospheres, which led to the formation of two kinds of SEI layers, consisting of Li_3_N (with high Li^+^ conductivity) in N_2_ and Li_2_CO_3_ and Li_2_O (with low Li^+^ conductivity) in N_2_ and CO_2_, respectively. Consequently, compared with a whisker-growth process in a CO_2_ environment, a surface-growth mode was observed in N_2_ via the facile surface transport of Li (Fig. 2d–f). The process of SEI formation was characterized by *in situ* transmission electron microscopy (TEM). Further exploration and discussion have also been held on the stress of multiphase SEI formation [24].

### Li-metal capacity degradation

In addition to the challenge of dendrite Li, the performance degradation due to the hyperactivity of Li metal is another hard nut to crack. There is a consensus that there is a protective SEI layer on the surface of Li-metal anodes, which originates from the reaction of active Li with the electrolyte. The continuous formation of an SEI layer and ‘dead Li’ consumes active materials during cycling, thus resulting in capacity loss.

#### The side reaction with electrolytes and the formation of an SEI

**Figure 3.**
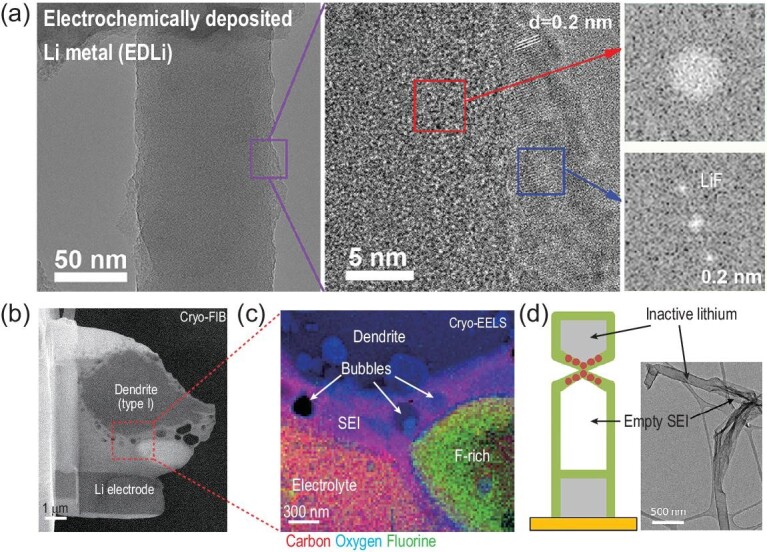
(a) Cryo-TEM image and its regional zoomed-in image with the bulk and surface area fast Fourier transform result of the electrochemically deposited lithium metal using conventional carbonate electrolyte [30]. Copyright 2017, American Chemical Society. (b) Electron transparent cryo-FIB lift-out lamellae [5]. Copyright 2018, Springer Nature Limited. (c) The electron energy loss spectroscopy (EELS) elemental mapping of regional zoomed-in image in (b). (d) Cryo-EM image and schematic of electrically disconnected and electrochemically inactive Li metal remains after full electrochemical stripping to 1.0 V [8]. Copyright 2018, Elsevier.

Li metal is able to react spontaneously with liquid electrolytes, including organic polar aprotic solvents, salt anions and additives. Indeed, the byproducts form a passivation layer on the surface of the Li-metal anode, which are generally insoluble lithium salts in the electrolytes. The ever-increasing passivation layer can block electrons and conduct lithium ions, which prevents further corrosion of the Li metal. However, the volume change and stress release during Li plating/stripping will break down the SEI layer and further consume active Li and electrolyte to form new passivation layers, finally resulting in low coulombic efficiencies (CEs). This kind of instantly formed passivation film is known as the SEI layer, which was first proposed in 1987 [25]. It is suggested that the characteristics of the SEI layer play a crucial role in the corrosion rate of Li metal, the mechanism of the Li-plating/stripping process, the kinetic parameters, the morphologies of the metal deposit and the half-cell potential. Therefore, the regulation of the SEI can be a good method for satisfying the performance of Li-metal anodes. The design of a desirable SEI layer has been generally employed to improve the stability and reversibility of Li-metal anodes by utilizing side reactions of Li metal with electrolytes. The development and application of advanced characterization techniques have contributed to confirming hypotheses and deepening the understanding of the formation and components of the SEI layer of lithium-metal anodes. Via *in situ* electrochemical transmission electron microscopy, it was confirmed that lithium plating occurs after the formation of SEI and remains on the surface of the electrode after Li stripping [26]. Furthermore, the sub-nanoscale resolution obtained by *in situ* scanning transmission electron microscopy (SEM) has revealed the fundamental mechanisms for SEI kinetics in Li-metal batteries, which include an inorganic–organic bilayer hybrid layer. This confirms the SEI growth at the SEI/electrolyte interface and indicates a radical species-associated (such as EC^–^ from ethylene carbonate (EC) or F^–^ from fluorinated ethylene carbonate (FEC)) growth mechanism for the SEI [27]. Atomic-resolution imaging of lithium metal shows great potential for revealing the structure and components of complicated SEI layers. However, high-resolution electron microscopy requires the use of high dose rates of electrons, which can damage the nanoscale structures of the SEI and Li-metal anode [28]. Fortunately, this dilemma has been solved by cryogenic (cryo)-electron microscopy, which has deepened the understanding of complicated nanoscale structures in batteries [29]. Based on cryo-electron microscopy, the crystallographic structure, growth direction and SEI nanostructures have been directly observed and recorded. The composition of the SEI has been revealed to consist of amorphous organic species and crystalline LiF (Fig. 3a) [30]. Moreover, two types of lithium dendrites associated with the SEI component have been identified via cryo-SEM [5]. Unexpectedly, dendrites consist of lithium hydride (LiH) rather than Li metal (Fig. 3b and c), which can contribute to the capacity loss of LMBs.

#### The formation of dead Li and Li-metal dusting

As early as 1977, some scientists proposed several possible mechanisms for capacity loss during cycling or an open-circuit state for LMBs [31]. They indicated that dendritic lithium is vulnerable to reaction with electrolytes, which yields insoluble and insulated products on the surface of the Li metal, until discontinuous electrical contact with the substrate. Consequently, the passivation layer (covering the Li granules) isolates the Li metal from participation in the charge–discharge process, leading to capacity fading of LMBs. This explanation for capacity degradation was summarized as the ‘dead Li’ mode [32]. Operando characterizations are necessary and beneficial to obtain a deep understanding, where these *in situ* techniques enable one to observe electrochemical processes directly and in real time, avoiding the possible evolution of components/morphologies/structures with changing environments and times. *In situ* SEM observations of the cross section of the battery can enable one to directly detect the morphology and indicate that Li deposits can evolve from a mossy-to-dendrite morphology at high current densities [33]. These dendrites are observed to be incompletely removed and stuck on the surface of the lithium (‘dead Li’). It should be noted that electron exposure can change the morphology of deposited Li, resulting in difficulties for *in situ* measurements that usually require a prolonged period of time [34]. To circumvent this issue, *in situ* environmental TEM has been used to investigate Li nucleation and growth. By using *in situ* environmental transmission electron microscopy, it has been indicated that the first dissolution of near-root segments leads to the formation of ‘dead Li’ in Li whiskers [35]. This explains why the newly formed SEI near the root of Li whiskers is much thinner due to the time lag, which leads to its dissolution prior to delithiation [35]. Moreover, cryo-electron microscopy reveals two types of SEI nanostructures (mosaic and multi-layer SEI), which are differentiated by the distribution of crystalline grains within the SEI [8]. It is worth noting that faster localized Li dissolution through high-crystallinity parts in the mosaic SEI results in notched structures during the Li-stripping process. Repeated cycling eventually leads to the formation of ‘dead Li’ (Fig. 3d). Recently, H_2_O titration and H_2_ gas chromatography were applied to quantify the content of unreacted metallic Li^0^ in components of the SEI [4]. The experimental results indicate that the formation of unreacted metallic Li^0^ dominates the CE loss. Meanwhile, the amount of Li^+^ in the SEI layers remains relatively constant in different electrolytes. These conclusions imply that the formation of ‘dead Li’ is responsible for the low initial CEs rather than the side reactions in different electrolytes.

**Figure 4.**
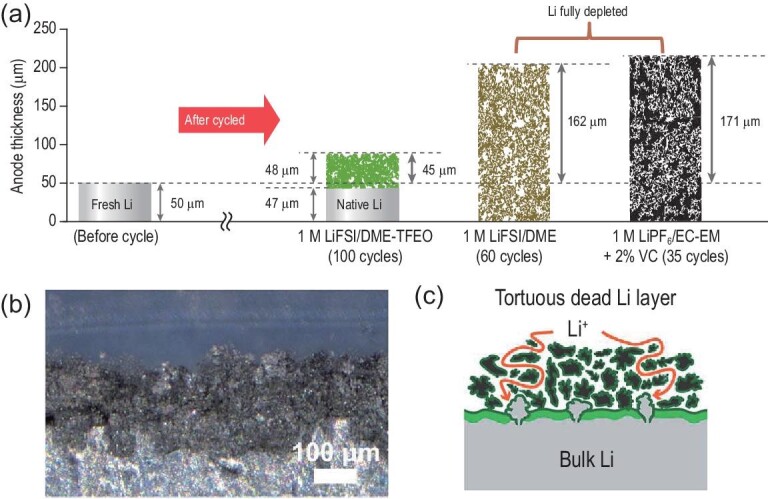
(a) Schematic of Li loss and corresponding thickness (volumetric) expansion after several cycles in different electrolytes [39]. Copyright 2019, Springer Nature Limited. (b) and (c) Cross-sectional operando microscopy images of the interphase and corresponding schematic representation of Li-ion diffusion show that a tortuous pathway is present after the accumulation of a thick dead Li layer on the electrode surface [38]. Copyright 2017, Royal Society of Chemistry.

In addition to dead Li, metal dusting is also a challenge, which is usually defined as the disintegration of metallic materials into dust composed of graphite and metal particles in the field of metallic material corrosion [36]. Li-metal dusting exists during lithium plating/stripping in the operation of Li-metal batteries. However, the reason and forming conditions are quite different from metallic material corrosion. Li-metal dusting results from the formation and accumulation of ‘dead Li’ (i.e. electrically isolated Li) through the porous SEI layer in non-aqueous electrolytes. The redissolution process continues to consume fresh Li and new Li sources and then redeposits within the porous interface, resulting in the accumulation of a dusting porous SEI layer [37] (Fig. 4a). Due to a tortuous pathway for Li-ion transport, the impedance of LMBs is thereby increased [38]. As a consequence, the increase in inner resistance has a negative impact on the total overpotential and the consumption of Li electrodes results in capacity loss (Fig. 4c and d), eventually leading to LMB failure.

It should be noted that Li-metal dusting here is confined to describe the failure mode of the Li anode. It is also called the Li-pulverization problem [39], the bulk Li-metal anode [40] and a corrosion layer [41]. In addition, dusted Li metal results in large volume fluctuations and shape changes during lithium stripping and plating. There is also reserved opinion on the correlation between Li-metal dusting and battery failure because no solid evidence has yet been found to support this augmentation. It has been proposed that dendrites in their test conditions lead to short circuits and cell failure [42]. Moreover, it is also indicated that a high-quality SEI layer can shield the process of Li-metal dusting against electrolyte attack, thus backing up its irrelevance to failure [41]. The influence of Li-metal dusting on capacity and failure was deeply analysed by understanding its mass transport effect [38]. The tortuous interphase from Li-metal dusting has an adverse effect on mass transport (i.e. lithium ions and anions). Therefore, a larger concentration gradient is formed to sustain Li-ion transport and the charge balance in the discharging process, resulting in an even higher overpotential. Consequently, it is easier to reach the fixed cut-off voltage, therefore leading to capacity loss for the cathodes. Based on the above analyses, Li-metal dusting emerges as dendrite growth occurs, which leads to Li-metal exhaustion, increases the inner resistance and degrades the cathodic capacity of LMBs.

## THE PRACTICAL SOLUTIONS AND APPLICATIONS OF L$\bf i$ METAL

The failure mechanism and mode reviewed above offer a better understanding of Li-metal anodes during electrochemical processes. This assists in providing guidelines for developing modification strategies and simultaneously paves the way toward developing safe and high-performance Li-metal anodes. Following this logic, many efforts have been made to fabricate Li-metal anodes to meet the high demand from practical solutions and to help realize excellent performance. According to the preparation methods and related applications, we herein classify Li-metal anodes into five different types and provide a detailed discussion about their unique characteristics in LMBs. Specifically, the five kinds of Li-metal anode are (i) SLMP, (ii) SLMA, (iii) DLMA, (iv) CLMA and (v) AFLMA.

### SLMP

**Figure 5.**
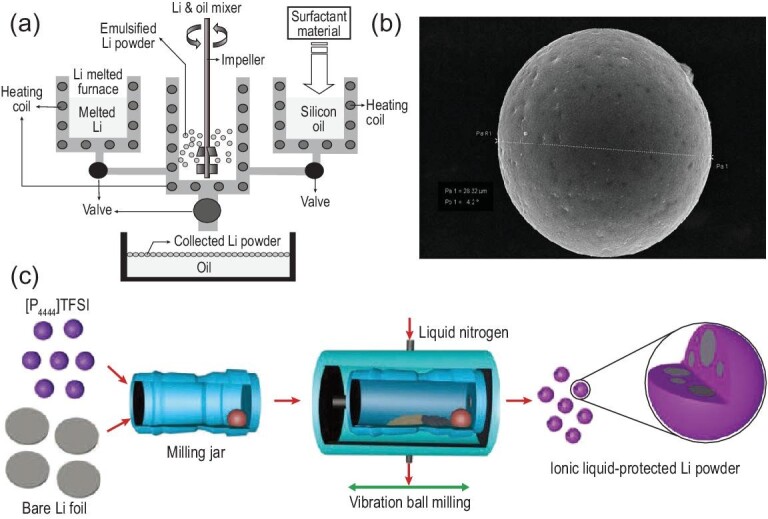
(a) Schematic diagram of the droplet emulsion technique (DET) apparatus [46]. Copyright 2004, Elsevier. (b) SEM image of single spherical SLMP particle [47]. Copyright 2011, Elsevier. (c) Schematic illustration of preparation process of cryomilling [48]. Copyright 2019, Wiley-VCH GmbH, Weinheim.

SLMP can compensate for the irreversible capacities of various anodes, which shows potential advantages of improving the capacity and initial CE in traditional LIBs and alternative energy-storage systems [43,44]. As shown in Fig. 5a, the production of SLMP is generally realized by a droplet emulsion technique (DET) [45,46]. A mixture of molten Li metal and an inorganic carrier fluid (i.e. silicon oil dissolved with surfactant materials) is sheared to generate an emulsion by high-speed dispersion at 20 000–25 000 rpm using DET equipment followed by a cooling and solidification process [46]. After that, uniform SLMP is obtained after separation from the carrier fluid and hexane washing. With the development of production techniques, SLMP has already been commercialized by the FMC Lithium company. It should be noted that the components (such as LiF and Li_2_CO_3_) and thickness of the protective coating layer vary according to the processing time and surfactant materials [46]. The typical morphology of commercialized SLMP is spherical particles with a controlled diameter (approximately 10–30 μm) and surface area, as shown in the SEM image of Fig. 5b. Commercialized SLMP consists of 97% wt. atomic lithium and a 3% wt. homogenously coated protective layer (i.e. Li_2_CO_3_), with a thickness of 100–1000 nm [47]. More importantly, it can be operated stably in dry air, which allows electrode slurry mixing for traditional film casting. Furthermore, SLMP can be easily and uniformly distributed in electrode slurries and as-prepared electrodes, with a well-controlled quantity of lithium powder [47]. However, the DET process suffers safety issues from the high reactivity of molten Li metal. As a coping strategy, a safer and more accessible cryomilling technology for the production of SLMPs was developed, as shown in Fig. 5c [48]. Liquid nitrogen is employed to create a cryogenic temperature, where soft and sticky Li metal becomes hard, brittle and easily processable. Then, a high-melting-point ionic liquid is used as a milling assistor and dispersion agent during the cryomilling process. The average diameter of SLMP particles obtained via the cryomilling process is ∼500 nm, which display improved electrochemical performance and great potential as a lithium source for the pre-lithiation of irreversible cathode/anode materials.

In general, the practical application of SLMP can be divided into two methods: one is to process SLMP into electrodes as anodes in LMBs and the other is to add them into electrodes to compensate for the irreversible capacities in LIBs. The SLMP electrode (consisting of compacted Li powders with an average diameter of 20 μm) display a 4.5-times larger surface area than the 2D lithium-metal foil, which facilitates reduction of the practical local current density during Li plating and stripping [46]. In addition, the well-designed protective layer with LiF or Li_2_CO_3_ can help control Li-dendrite growth, which has been confirmed by using an *in situ* optical cell [49]. Meanwhile, SLMP can also act as a candidate pre-lithiation source for cathode (such as Li-free cathode) and anode materials, with huge irreversible capacity loss in the first few cycles [44,50]. Moreover, SLMP can be used not only in LMBs but also in LIBs. This can offset the irreversible capacities of cathode and anode materials for LIBs. Only a relatively small quantity of SLMP is required to compensate for the depletion during the initial formation, ensuring no residual LIBs, as confirmed by ^7^Li NMR [51].

### SLMA

The lithium-metal anode was commercialized and widely used in half cells ∼30 years ago. Explorations of SLMAs, including study of the stabilized electrode/electrolyte interface and adjustment of electrolyte components, greatly facilitate the understanding and improvement of LMBs. As discussed above, fresh lithium metal faces two serious problems of dendrite growth and Li-metal dusting, which cause performance degradation and even short circuits of cells. As a result, most relative strategies aim to solve these problems, especially the poor stability of Li-metal electrode/electrolyte interfaces. These modification methods include constructing artificial SEI layers (organic, inorganic or hybrid layers), optimizing electrolyte composition (i.e. solvents, Li salts, additives), using alloying processes and designing new separators. The evaluation standards for SLMA focus on the stability in symmetric cells and the capacity retention in full cells.

**Figure 6.**
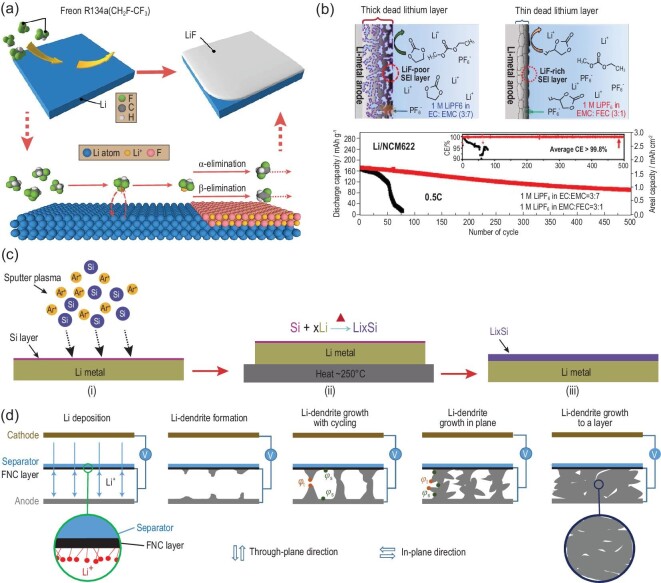
(a) Schematic diagrams of surface LiF coating and major chemical reactions at the early stage [53]. Copyright 2017, American Chemical Society. (b) Schematic diagrams and cycling performance of the Li|NCM622 cell in different electrolytes [63]. Copyright 2018, American Chemical Society. (c) The preparation process of Li_x_Si-modified lithium foil [64]. Copyright 2018, Wiley-VCH GmbH, Weinheim. (d) A FNC cell using a FNC-coated separator [65]. Copyright 2017, Springer Nature Limited.

The SEI plays an important role in the high CE and long cycle life of LMBs. The SEI layer generally results from the side reaction of hyperactive Li metal with non-aqueous electrolytes. However, the spontaneously formed SEI layer is usually fragile and easily cracked by local stress from non-uniform Li plating and stripping, leading to repeated lithium depletion [52]. The properties of an ideal SEI layer include high Li-ion conductivity, homogeneous chemical composition, high chemical/electrochemical stability and suitable mechanical strength [11]. Consequently, a well-designed artificial SEI layer paves the way toward a SLMA with minimized Li-metal dusting (Fig. 6a) [53]. Lithium-ion solid electrolyte materials are considered the most promising candidates for stabilizing the electrode–electrolyte interface and protecting lithium-metal anodes, which has been previously summarized in other literature [54]. This has opened up a broad strategy for selecting suitable solid-state electrolytes and constructing excellent artificial SEI layers on Li-metal surfaces. As a result, various kinds of lithium-ion conductors have been used as artificial SEI layers for Li-metal anodes, such as Li_3_N [55].

Liquid electrolytes are the most important component of non-aqueous Li-ion batteries, which are usually composed of solvents, lithium salts and additives [56]. The formation of an SEI and CEI (cathode electrolyte interphase) is greatly related to the electrolyte decomposition, including chemical/electrochemical reactions [57]. As a result, the adjustment of the electrolyte component plays a crucial role in stabilizing the lithium-metal anode. The morphology and cycling performance of Li plating in various kinds of Li salts and solvents were systematically explored and studied, and it was found that a glyme family (dimethoxy ethane (DME, CH_3_OCH_2_CH_2_OCH_3_), ethyl glyme (EG, CH_3_CH_2_OCH_2_CH_2_OCH_2_CH_3_) and diglyme (DG, CH_3_OCH_2_CH_2_OCH_2_CH_2_OCH_3_)) shows much less reactivity than cyclic ethers, esters and alkyl carbonates [58]. Consequently, DME has been widely used as a solvent for stabilized Li-metal anodes. However, the oxidation stability of ether-based solvents with a salt concentration of 1 M (1 mol/L) has been proven to be <4 V versus Li/Li^+^, which severely limits their application for high-voltage cathode materials [59]. As a result, ether-based solvents, such as DME, are mainly used for low-voltage battery systems: Li–S, Li–lithium titanate (LTO) and Li–lithium iron phosphate (LFP). It has been reported that a high salt concentration (solvent in salt) in ether-based solvents (1,3-dioxolane (DOL)/1,2-dimethoxyethane (DME)) can suppress the dissolution of lithium polysulfide and metallic lithium dendrite growth [60]. More importantly, it was proven that a high salt concentration can generate an effective CEI through synergy of the salt and ether solvent [61], which helps stabilize high-voltage electrode materials. In the promotion of highly concentrated ether-based electrolytes, a dual-salt system was further developed to stabilize the SEI for Li-metal anodes and the CEI for high-voltage cathodes at the same time [41]. It is worth noting that additives have also played a vital role in the development of electrolytes for stabilized Li-metal batteries [40]. Following investigation of the effect of hydrogen fluoride (HF) on Li metal, it was proposed that the formation of LiF can be attributed to a uniform current distribution [62]. Therefore, various kinds of fluoride additives have been developed to stabilize the SEI on the surface of Li metal, such as fluoroethylene carbonate (Fig. 6b) [63]. Considering the low oxidation stability of widely used ether-based electrolytes, new solvent systems are being explored to extend the potential window for high-voltage cathodes [39].

The alloying process has been applied to solve the problems of Li metal for several decades, such as Li–Al alloys. It is widely reported that alloying or lithium intermetallics can reduce the hyperactivity of Li metal and suppress its side reactions with liquid electrolytes. Various kinds of Li alloys have been explored and investigated to stabilize Li-metal interfaces with liquid electrolytes, such as Li–Si [64] (Fig. 6c). In addition, alloying strategies enable facile and applicable manufacturing of practical LMB anodes for mass production. In addition, to prevent the formation of electrical short circuits due to dendritic Li penetration, separators are used as the last defense for LMBs. The modification of separators for LMBs has generally focused on the suppression of Li-dendrite penetration and the homogenization of the lithium-ion flux [65] (Fig. 6d). It can be anticipated that the progress for SLMA paves an accessible way to evaluate the effects of electrolytes, separators and cathodes. However, the amount of lithium-metal anode is excessive compared to cathode materials, which will weaken the influence of Li-metal depletion on performance degradation [66]. As a consequence, SLMAs are far from practical applications.

### DLMA

**Figure 7.**
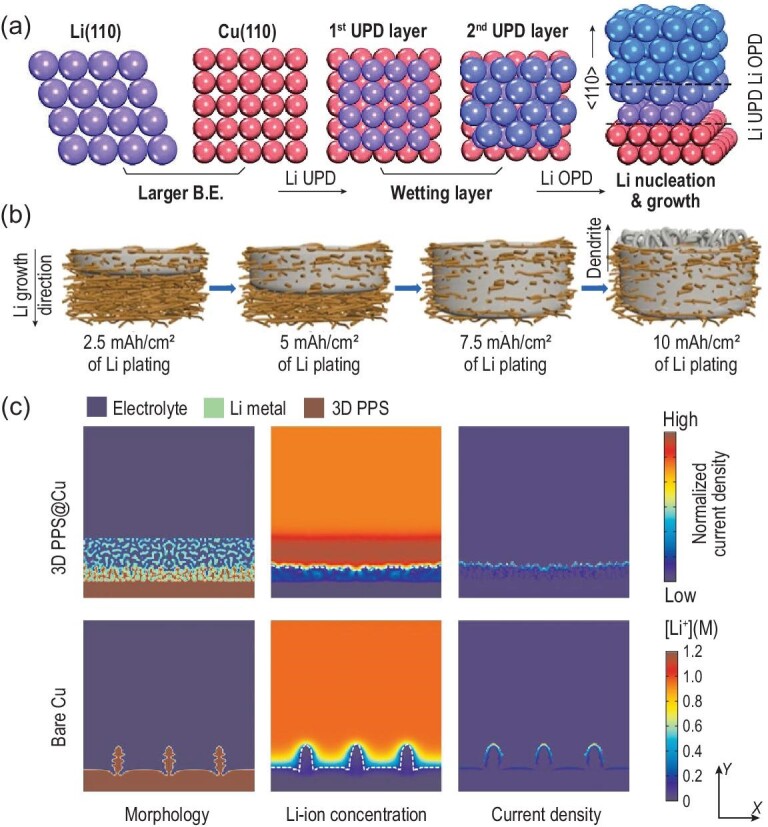
(a) Schematics of the structures of Li UPD layers and the crystallographic orientation of deposited bulk Li [70]. UPD, underpotential deposition; OPD, overpotential deposition. Copyright 2019, Wiley-VCH GmbH, Weinheim. (b) Schematic illustration of the Li plating in the 3D CuNWs network current collector at different statuses [78]. Copyright 2016, American Chemical Society. (c) 2D phase-field simulation of Li deposition on 3D PPS@Cu and bare Cu electrodes [72]. PPS, porous polyethylenimine sponge. Copyright 2018, Springer Nature Limited.

A DLMA provides an effective method to test the CE and evaluate the depletion of active Li during the plating/stripping process in half cells. The controllable areal capacity of deposited Li is suitable for the evaluation of stability, while the predeposition process generally requires complicated operation (i.e. disassembly and reassembly of Li/Cu half cells). The study of DLMA is similar to that of SLMA, although DLMA is much more hyperactive than a Li-metal foil/plate (generally with a passive layer) [67]. The optimization of electrolytes and separators for SLMA discussed above is usually applicable for deposited LMB systems [68,69]. Limited by the hyperactivity and low melting point of Li metal, modification methods for Li-metal foil are generally performed under moderate reaction conditions. Apart from the modification strategies used for SLMAs, more powerful methods have been proposed to tackle Li-metal problems, such as constructing lithiophilic structures, 3D current collectors and 3D electron-insulated skeletons [70–72]. These methods focus on the adjustment and promotion of the current collectors, thus realizing uniform Li deposition with suppressed dendrite growth. Designing a lithiophilic structure on the current collector is widely considered a powerful method to regulate Li-deposition sites and behavior [73]. Copper foils are widely applied as a general current collector for Li deposition. However, the lattice of pristine copper (Cu) foil exhibits a huge thermodynamic mismatch with Li metal, leading to a huge nucleation barrier (i.e. nucleation overpotential during the Li-plating process) with non-uniform Li plating [74]. Li metal prefers to deposit onto the Cu (100) face with a lower nucleation barrier, whose lithiophilicity arises from surface lattice matching with the Li (110) face (Fig. 7a) [70]. In addition, the alloying process and the lithophilic/lithiophobic (or anionic) structure are widely used to eliminate the lattice mismatch with deposited Li metal and control its deposition site (such as selective Li plating into a hollow carbon sphere with metallic nanoparticles) [74–76]. Uniform Li-plating/stripping behavior is obtained through lithiophilic optimization, accompanied by a dendrite-free and smooth morphology for the deposited Li.

Under low-current-density conditions, diffused Li ions compensate for consumed Li ions in the process of Li deposition and result in dendrite-free deposited lithium [77]. A large specific surface area for a 3D current collector enables one to decentralize the applied areal current at a high current density and realize a relatively low practical local current, which helps regulate and homogenize Li-plating/stripping behavior [77]. Moreover, the porous structure of well-designed 3D conductive structures provides enough space for accommodating deposited lithium and releasing the volume change generated by Li plating/stripping [78] (Fig. 7b). A large variety of 3D conductive structures have been developed for depositing lithium-metal anodes, such as 3D copper structures [71] and 3D Ni foams [79]. However, without additional treatment, conductive surfaces generally suffer from preferential lithium nucleation and dendritic growth. To tackle this issue, the treatment of a conductive separator-facing surface has been proposed to build a nonconductive layer (such as SiO_2_ and by-produced SiC) [80]. In addition, 3D conductive current collectors with lithiophilic design enable preferential Li plating on the away-facing separator surface, which improves the utilization of interior spaces and avoids the problem of Li dendrites on the separator-facing surface [81]. As a result, the areal capacity and current density are highly improved at the same time without dendritic Li formation.

A 3D electron-insulated skeleton structure is another solution for solving the problem of dendritic Li. There are a few advantages in this configuration:

first, Li ions can only gain electrons and become metallic Li^0^ at the bottom of the conductive substrate instead of the nonconductive upper surface of the electrodes; second, these nonconductive structures with strong Li-ion interaction/affinity

(Fig. 7c) (such as a 3D polymer sponge [72]) are able to redistribute Li ions and tackle the problem of dendrite growth due to concentration polarization; third, the void space of the 3D electron-insulated skeleton can accommodate deposited lithium and relieve volume changes [80]. Although electrolyte optimization plays a crucial role in Li-metal anodes, the deposited lithium metal has higher hyperactivity than the stabilized lithium metal; thus, ether-based solvents (such as DME) dominate the liquid electrolytes in pre-deposited Li-metal anodes. Furthermore, high salt concentrations, dual-salt/ternary salt systems, alternative solvents, Li salts and additives have been widely explored to stabilize deposited lithium and improve cycling efficiencies [68,69,82,83]. Notably, the CEs of deposited Li metal (generally <99.5%, as shown in Table 1) are still lower than that of commercial graphene materials (close to 99.9%). Even if the average CEs reach 99.5% in the first 100 cycles, 40% of the Li source will be consumed in the cathode materials. In addition, the laboratory-level process is complicated and not suitable for the factory-level mass production of pre-deposited Li metal. Limited by the pre-lithiation process, deposited lithium-metal anodes are far from final practical application. The application of commercial stabilized lithium-metal powders for the pre-lithiation of the above well-designed current collectors in deposited lithium-metal anodes shows great potential. Corresponding progress guides the research into high-energy LMBs for the next generation.

**Table 1.** Performance comparison of deposited lithium-metal anode (DLMA).

Coulombic efficiency	Cycle number	Testing condition	Anode	Electrolyte	Reference
99	200	4 mAh cm^–2^ at 2 mA cm^–2^	Carbon-coated 3D Ni foam	1 M LiTFSI DOL/DME (1 : 1 w/w) 1 wt% LiNO_3_	*Chem Commun* 2018; **54**: 5330 [79]
99.1	100	1 mAh cm^–2^ at 1 mA cm^–2^	Au-modified carbon paper	1 M LiTFSI DOL/DME (1 : 1 v/v) 1 wt% LiNO_3_	*Energy Storage Mater* 2019; **16**: 259–66 [81]
99.1	450	1 mAh cm^–2^ at 0.5 mA cm^–2^	Cu foil	1 M LiTFSI DOL/DME (1 : 1 v/v) 3 wt% LiNO_3_, LiFSI : LiTFSI = 2 : 1(molar ratio)	*Adv Energy Mater* 2018; **9**: 1803372 [68]
99.6	500	0.5 mAh cm^–2^ at 0.5 mA cm^–2^	Cu foil	0.3 M LiTFSI and 0.3 M THF in CH_3_F	*Joule* 2019; **3**: 1–15 [69]
99.3	300	1 mAh cm^–2^ at 1 mA cm^–2^ (60^o^C)	Cu foil	1M LiTFSI DOL/DME (1 : 1 v/v) 1 wt% LiNO_3_	*Nat Energy* 2019; **4**: 664–70 [17]

### CLMA

CLMAs are generally composed of conductive structures and lithium metal, which take advantage of the DLMA and avoid the problems of complicated predeposition processes. The preparation of CLMAs involves two main kinds of methods: the molten-lithium method and the mechanical rolling method, both of which show greater potential for mass production compared to deposited lithium-metal anodes.

**Figure 8.**
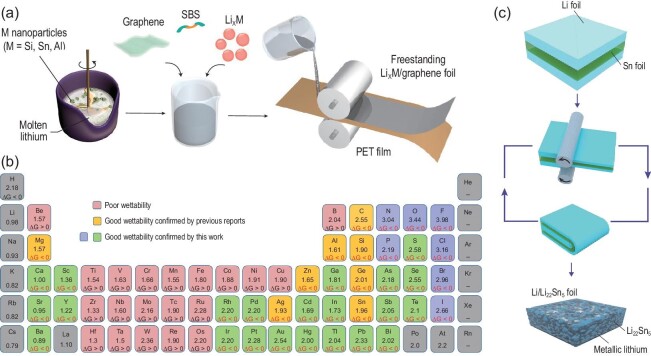
(a) Schematic of fabricating the freestanding Li_x_M/graphene foil [85]. Copyright 2017, Springer Nature Limited. (b) Electronegativities of various elements in the periodic table and ΔrG of elements or compounds reacted with the molten Li, which is responsible for the improved wettability [84]. Copyright 2019, Springer Nature Limited. (c) Schematic of the fabrication of the Li/Li_22_Sn_5_ nanocomposite foil [90]. Copyright 2020, Springer Nature Limited.

The wettability with different substrate materials is fundamental for molten-lithium preparation. However, molten lithium shows poor wettability on pristine copper substrates [84]. In addition, poor lithiophilicity is also observed on the surface of 3D foamed copper, 3D foamed iron, 3D foamed nickel, carbon fiber and oxidized graphite [84]. These 3D conductive structures are widely used for deposited lithium metal, but they may not be suitable for the preparation of composite lithium metal via the molten method. Consequently, modified 3D conductive skeletons with lithophilic treatment (Fig. 8a) have been widely reported to assist the wetting process for liquid molten lithium in recent years [85,86]. It is worth noting that by using the molten-lithium method, a specific CLMA can show practical stability in air [85]. A crucial technique to fabricate the composite involves the wettability between the molten liquid Li and matrix. During investigation into the wetting property of molten lithium, it was suggested that high temperature can reduce the contact angle with substrates [87]. The wettability of substrates can be improved by coating, which is driven by the negative Gibbs free energy of the chemical reactions that occur between the coating materials and liquid molten lithium. Coating carbon scaffolds with lithiophilic silicon has been reported for lithium as a CLMA to create lithiophilic surfaces and acquire improved wettability [37]. Lithium can fill the empty space between the carbon matrix quickly and make the whole anode mechanically and chemically stable during operation. Experiments have also revealed that graphite can be superlithiophilic or lithiophobic depending on its local redox potential and thus influence the wettability of the corresponding substrate [88]. It was found that lithiated porous carbon paper has improved wettability with lithium and, following this trait, a Li-graphite composite anode with a specific Li/C ratio was prepared [88]. It should be mentioned that the formation of Li_2_O on the surface of molten lithium results in a kinetic barrier for lithium spreading and wetting. In addition, superlithiophobic materials with rough surfaces are proposed to guide electrode design, similar to water wetting. Systematic exploration into the wettability of organic coatings on copper foil and element additives in molten lithium toward the preparation of ultra-thin lithium-metal anodes reveals that the formation mechanism for lithiophilic substrates is mainly related to the formation of new chemical bonds with molten lithium [84]. A general list of lithiophilic elements has been confirmed with the evaluation of Gibbs free energy values, which has never been previously used for the lithiophilicity of molten lithium (Fig. 8b).

Although molten-lithium preparation has proven to be a powerful method for achieving high-performance lithium metal, its higher reactivity requires stricter conditions than lithium-metal solid foil. Consequently, the mechanical processing for lithium-metal foil is considered to be a facile and low-cost preparation method for CLMAs. The mechanical rolling process has been used to prepare 2D metal nanolayer structures [89]. Inspired by the progress reviewed in the section for deposited lithium-metal anodes, the mechanical rolling process has also been applied to construct CLMAs, such as preparing 3D Li/Li-Sn alloys (Fig. 8c) via a rolling/repeated stacking approach [90].

Based on the advantages and progress of deposited lithium-metal anodes, a general method was proposed to construct CLMAs with modified surfaces [91]. 3D Cu_3_N nanowires (NWs) were directly rolled onto lithium-metal foil and the formed Li_3_N@Cu NW layer was found to display high Li-ion conductivity, thermodynamic stability and lithiophilicity. This kind of mechanical process opens up a new way toward realizing facile surface treatment for advanced lithium anodes. In addition, the CLMA prevents the robust problems of dendritic growth and capacity loss in pristine lithium-metal foil and the complicated pre-lithiation process of deposited lithium metal. Accompanied by facile preparation, CLMAs have become the most promising candidate for mass production and application in LMBs [92]. Specifically, CLMA in solid-state LMBs offers high energy density and high safety. It is known that the important issue to be solved in solid-state batteries is the insufficient contact at the interface of the lithium anode and solid electrolyte. CLMAs can be used to improve the adhesiveness of Li metal to solid-state electrolytes by using Li-alloy anodes or surface coatings for *in situ*-formed lithiophilic interfaces [93,94]. For example, Cu_3_N can be mixed with molten Li to form Li_3_N, which can create better contact with the electrolyte and improve the interface between the two materials [92]. Hexagonal boron nitride (h-BN) is also an ideal modifier for stabilizing the Li-metal interface with Li-metal–BN nanosheet composite anodes and shows high adhesiveness for solid-state LMBs with garnet electrolytes. Moreover, the composite and *in situ*-formed Li_3_N at the interface can effectively suppress Li dendrites owing to the feature of an electrical insulator that isolates electrical contact between Li and garnet [94]. Enhanced contact at the interface offers a high critical current density of 1.5 mA cm^–2^ and stable operation for the battery, which indicates that the composite anode might offer a candidate solution to the interfacial issue for solid-state LMBs.

### AFLMA

**Figure 9.**
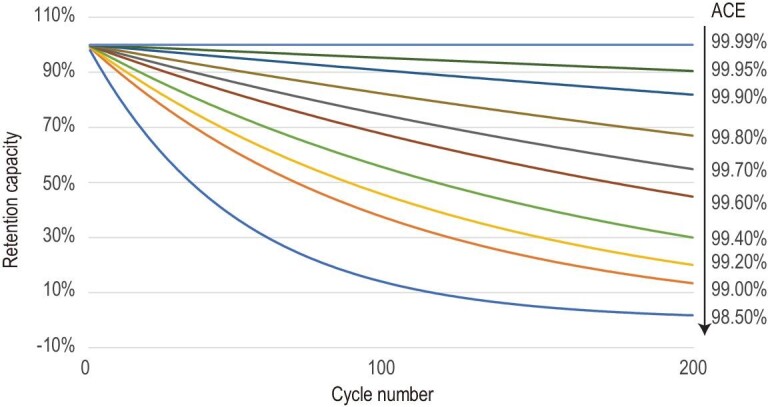
Retention capacity of anode-free lithium-metal cells at different average coulombic efficiencies.

The complicated pre-lithiation process for deposited lithium-metal anodes can be circumvented by using an AFLMA strategy (such as bare copper foil). In such batteries, Li ions are extracted from the cathode and plated onto the Cu current collector during the charge process. Ideally, Li^+^ can be reversibly stripped from the Cu foil and intercalated into the cathode. The battery is initially assembled without an active anode material and is therefore defined as an anode-free lithium-metal battery (AFLMB). In addition to easy assembly and reduced cost, AFLMBs also lead to a significantly increased energy density owing to the reduced weight and space of anodes such as graphite. Moreover, the theoretical capacity of the Li anode is much higher than that of the commonly used graphite anode (3820 mAh cm^–2^ for Li versus 372 mAh cm^–2^ for graphite). In addition, the AFLMA offers a higher operating voltage of ∼0.1 V than a graphite anode, which results in ∼60% more energy per volume (Wh L^–1^) than conventional LIBs. Specifically, on the basis of the maximum 400 km possible for present EVs, an additional 280 km can be expected with such an increase [93]. Despite these advantages, AFLMBs suffer from a rather limited cycle life (generally a capacity retention of 80% after <20 cycles) owing to great challenges, including low CE and the growth of dendritic Li [93]. The former can consume the active Li from the cathode within a few cycles, which is a result of the reactivity between the Li and electrolyte. The dendrite, meanwhile, can even lead to penetration of the separator and short-circuiting of the battery. Previous reports generally show an average CE of <99.6%, which means that only 44.86% of the lithium source in cathode materials remains after 200 cycles in an anode-free lithium-metal battery. Subsequently, the retention capacity for anode-free LMBs is calculated based on different average CEs (Fig. 9).

In fact, the capacity retention of LMBs can be artificially inflated by excess lithium, even at an average CE of <99%. As a comparison, anode-free LMBs are a facile and powerful tool for the evaluation of performance degradation and promotion strategies. The main idea in this strategy still focuses on the modification of electrolytes, as discussed in the previous sections. However, most reports show rapid capacity degradation to <80% after several cycles in anode-free cells [94]. In short, advances in alternative solvents and Li salts can inspire and motivate the study of anode-free lithium-metal cells [95,96]. Herein, we summarize and compare the cycle performance of three typical anode-free lithium-metal cells, as shown in Table 2. It has been confirmed that the consumption of lithium includes the formation of dead Li (i.e. unreacted metallic Li^0^) and the SEI Li^+^ amount. The SEI Li^+^ amount dominates the capacity loss when the CE is >95%. As a result, great improvements in the CEs should focus on decreasing the accumulation of dead Li and the further formation of an SEI. A dense morphology of deposited lithium was observed by studying the effect of external pressure on cyclability [97]. External stack pressures accompanied by a dual-salt strategy were then applied to suppress the formation and accumulation of dead Li and prevent the further corrosion of deposited Li in liquid electrolytes, leading to excellent performance in anode-free lithium-metal pouch cells [98].

**Table 2.** Performance comparison of anode-free lithium-metal anode (AFLMA).

Cathode	Anode	Electrolyte	Retention capacity	Reference
LiNi_0.6_Mn_0.2_Co_0.2_O_2_ (1.44 mA h cm^–2^)	Cu foil	4.6 m LiFSI + 2.3 m LiTFSI in DME	55% after 54 cycles	*Energy Environ Sci* 2019; **12**: 780–94 [96]
LiNi_0.8_Mn_0.1_Co_0.1_O_2_ (4.2 mAh cm^–2^)	Cu foil	LiFSI-1.2DME-3TTE (in molar ratio)	77% after 70 cycles	*Joule* 2019; **3**: 1662–76 [95]
LiNi_0.5_Mn_0.3_Co_0.2_O_2_ (2.4 mAh cm^−2^)	Cu foil	0.5 m LiDFOB + 0.5 m LiBF4 in FEC: DEC (1 : 2 (v : v))	80% after 90 cycles	*Nat Energy* 2019; **4**: 683–9 [98]

**Figure 10.**
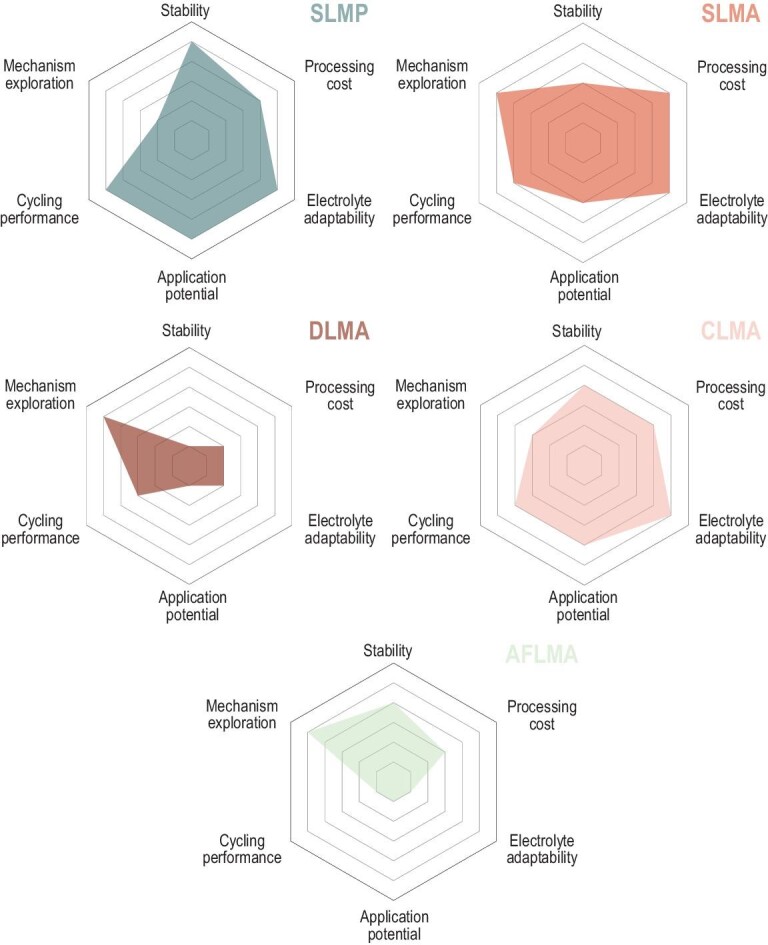
Performance comparison of five kinds of lithium-metal anode proposed in this review. Radar plots of the performance properties of stabilized lithium-metal powder (SLMP), stabilized lithium-metal anode (SLMA), deposited lithium-metal anode (DLMA), composite lithium-metal anode (CLMA) and anode-free lithium-metal anode (AFLMA).

As shown by comparison in Fig. 10, SLMP shows great practical potential for compensating for the irreversible capacity loss of commercial anode materials, such as graphite. The main modified targets for SLMAs are the reduction of Li-metal dusting and the suppression of Li-dendrite growth. A DLMA allows the modification of the current but requires complicated predeposition of Li and disassembly of batteries. A CLMA takes advantage of the design of suitable electrode structures and avoids the complicated process of Li predeposition. An AFLMA employs bare Cu foil as an anode, which avoids the complicated Li predeposition process. Among them, a CLMA is suggested to be the most promising candidate for the commercialization of LMBs in the future. SLMP is projected to play an enormous role in the promotion of energy density for LIBs in the near future. For the other three types of lithium anodes, the corresponding theoretical and experimental exploration has deepened the understanding of the Li-metal failure mode and promoted strategies for future practical applications.

## CONCLUSION AND OUTLOOK

In this review, we have summarized recent insights into the failure mechanism for lithium-metal anodes in non-aqueous electrolytes, followed by the proposal of two failure modes: one mode involves sudden short-circuiting due to dendrite formation, with the growth of lithium dendrites arising from concentration polarization and stress release; the other failure mode involves the slow process of capacity degradation, accompanied by increased inner resistance originating from the SEI and the accumulation of dead Li. Benefitting from the combination of advanced characterization methods and *in situ* characterization technologies, some hypotheses have been verified recently and new theories have been proposed. According to the preparation method and application potential, five types of lithium-metal anodes are classified and summarized: SLMP, SLMA, DLMA, CLMA and AFLMA. However, although a deeper understanding of Li-metal anodes has been developed, there is still a long way to go for available LMBs. No commercial lithium-metal battery with high safety has been produced and commercialized thus far. The ultimate form of lithium-metal anodes remains uncertain, despite the many optimized lithium-metal anodes discussed in this review. In addition to the Li metal itself, modification of the electrolyte should also be investigated and well designed for various Li-metal anodes. Therefore, more attempts and efforts need to be made to find solutions for the use of lithium anodes for practical high-energy LMBs.
